# Anatomic parameters of omphaloceles and their association with anatomic, genetic, or syndromic malformations: a retrospective study

**DOI:** 10.1007/s00383-024-05717-w

**Published:** 2024-05-23

**Authors:** Gallien Parata, Yvan Vial, Marie-Claude Addor, Jean-Marie Pellegrinelli, Barbara E. Wildhaber

**Affiliations:** 1 Service médico-chirurgical de pédiatrie, Hôpital du Valais, Sion, Switzerland; 2 Service of Obstetrics, Department of Woman-Mother-Child, University Medical Centre CHUV, Lausanne, Switzerland; 3 Service of Genetics, Department of Woman-Mother-Child, University Medical Centre CHUV, Lausanne, Switzerland; 4 Division of Obstetrics, Department of Pediatrics, Gynecology, and Obstetrics, University Hospital of Geneva, Geneva, Switzerland; 5 Division of Child and Adolescent Surgery, Department of Pediatrics, Gynecology, and Obstetrics, Geneva University Hospitals, University of Geneva, 6, Rue Willy Donzé, 1205 Geneva, Switzerland

**Keywords:** Omphalocele, Classification, Anomalies, Defect, Size

## Abstract

**Purpose:**

This retrospective study aims to describe anatomical parameters of omphaloceles and to analyze their association with anatomical, genetic, or syndromic malformations.

**Methods:**

Cases were selected from digital records of two university centers, a certified regional registry and personal records. Patients from 1998 to 2018 with omphalocele and live birth (LB), termination of pregnancy due to fetal anomaly (TOPFA) and fetal death (FD) were included. Cases born outside Western Switzerland and/or with upper or lower coelosomy were excluded.

**Results:**

We analyzed 162 cases with the following distribution: 57 (35%) LB, 91 (56%) TOPFA and 14 (9%) FD. TOPFA was significantly more frequently performed in cases with non-isolated omphalocele, i.e., omphaloceles with associated major malformations (especially cardiovascular and genitourinary), genetic/chromosomal anomalies, or syndromes. For LB, associated anatomical malformations, genetic or chromosomal anomalies were not significantly associated with the size of the omphalocele or the liver involvement.

**Conclusions:**

The proportion of cases resulting in TOPFA was higher among fetuses with major malformations, genetic or chromosomal anomalies. Despite the large size of this cohort, and in contrary to previous publications, the size of the omphalocele and/or liver involvement does not allow for conclusions regarding the presence or number of associated malformations, genetic or chromosomal anomalies.

## Introduction

Omphalocele is a congenital abdominal wall defect defined by herniation of viscera through the umbilicus into the umbilical cord. The organs are covered by a membrane built by the peritoneum, Wharton’s jelly and amniotic membrane [1]. Total prevalence of omphalocele in Europe over the period 1998 to 2018 is 3.37/10,000 and live birth prevalence is 1.24/10,000 according to EUROCAT registry. Its prevalence does not seem to vary over time [2, 3]. Some risk factors for omphalocele development have been reported such as in vitro fertilization and young or advanced maternal age. Also, some familial cases have been described [4].

The etiology of omphalocele remains unknown, but it seems to be more associated with genetic factors rather than environmental factors. Studies in animals have identified genes like Rock-1, Pitx2, IgFr, Msx1, and Msx2 that may play a role in its development [5, 6]. Human and animal embryological studies suggest errors in the secondary formation of the abdominal wall or non-reintegration of the physiologic hernia before the 10th week of gestation as potential explanations [7]. These modern explanations differ from the original proposal by Duhamel in 1963, which suggested non-fusion of the lateral folds [6, 8, 9]. The presence of various syndromes and associations with omphalocele suggest the possibility of different pathways leading to this malformation.

Omphalocele is typically diagnosed during the first or second trimester prenatal ultrasound. It is frequently associated with anatomical abnormalities and / or genetical and chromosomal anomalies including trisomy 13, 18, 21, Beckwith-Wiedemann syndrome and omphalocele-cloacal exstrophy-imperforate anus-spinal defect complex (OEIS complex) [10]. Thus, extensive prenatal investigations are required [11–13]. Depending on local policy and culture, an estimated proportion arising from 0 to 83% of pregnancies with babies presenting an omphalocele are medically interrupted [12–14].

In Switzerland, since 2002 the Swiss Penal Code strictly regulates pregnancy termination, allowing for voluntary termination up to the 12th week of amenorrhea, and at any point during pregnancy in case of serious fetal or maternal medical reasons [15]. Prior to 2002, abortion was criminalized by the law of 1942, with exceptions only in cases of maternal risk. Since the 1980s, this legislation has become obsolete, with the last judgment for this offense pronounced in 1988. Although legislation only changed in 2002, it appears from Federal Statistical Office data that since 2000 the practice and epidemiology of terminations has remained largely unchanged [16, 17]. Switzerland has a low overall rate of overall pregnancy termination compared to international standards, with 6.8 terminations per 1000 women in 2017 [17], compared to the world average estimated at 36 terminations per 1000 women and 16 terminations per 1000 women in Western Europe [18]. The proportion of *medical* terminations in Switzerland is 5% [17]. The Swiss Federal Statistical Office has been recording cases of pregnancy terminations since their legalization in 2002.

The presence and severity of associated malformations are known to be the strongest predictors of morbidity and mortality in omphalocele cases [10, 19]. However, there is no international consensus on the classification of omphalocele. Some classify it as minor, major, giant, or ruptured, with threshold ranging from 5 to 8 cm at different sites of the defect while others prefer descriptive criteria such as the possibility of reduction in a single operating time, or presence of the liver in the defect, making it challenging to compare different studies [20].

Recently, omphalocele description in absolute terms has been criticized. Since 2011, various prenatal ultrasonography studies have investigated the usefulness of ratios in predicting outcome and/or association with malformations: it has been proposed to normalize the size of the omphalocele to the length of the femur (OC/FL), to the abdominal diameter (OC/AD) or to the head circumference (OC/HC). Despite promising studies, results are contrasting and do not yet provide clear predictive criteria [21–23].

Several studies have investigated associations between the presence of malformations and omphalocele measurements: results are controversial and so far no consensus has been reached [24, 25]. In this study, we hypothesize that some descriptive anatomical parameters of omphaloceles are associated with a trend for anatomical, genetic or syndromic malformations.

This study aims to describe anatomical parameters of omphaloceles and to analyze their association with anatomical, genetic, or syndromic malformations. Our aim is to provide new data that will contribute to the establishment of a prognostic classification for omphalocele.

## Methods

We conducted a retrospective, observational study on omphalocele cases in Western Switzerland from 01.01.1998 to 31.12.2018. We defined Western Switzerland as the territory covered by the cantons of Geneva, Vaud, Neuchâtel, Jura, and Valais. Inclusion criteria were patients with the diagnosis of omphalocele, such as cases with live birth (LB), termination of pregnancy due to fetal anomaly (TOPFA) or fetal death (FD). The following databases were used to identify cases: i) institutional databases of the two university centers of Western Switzerland (Geneva University Hospitals, Lausanne University Hospital; ii) personal databases of practitioners in pediatric surgery, obstetrics and genetics, and iii) the local registry of Eurocat-Vaud Switzerland (the registry of Vaud-Switzerland, included in EUROCAT, the network of population-based registries for the epidemiologic surveillance of congenital anomalies in Europe). Exclusion criteria were umbilical hernia, upper and lower coelosomia, very poorly documented cases, and cases born outside of Western Switzerland.

After exclusion of duplicates and coding errors, we analyzed clinical documents such as clinical reports, operative reports, autopsy reports, or ultrasound reports for each case. The information collected included LB, TOPFA, FD, gestational age at birth, birth weight,.Prenatal and neonatal measurements (head circumference, abdominal diameter, femur length), presence of associated malformations, syndromes, or genetic/chromosomal anomalies, omphalocele measurements, and contents of the omphalocele (liver, intestines, others) were collected based on ultrasound reports and clinical reports. We classified omphaloceles as isolated (without any associated malformation or chromosomal abnormality) or syndromic (associated with Down syndrome, Patau syndrome, Edward’s syndrome, Beckwith-Wiedemann syndrome, or OEIS complex). Prematurity was defined as birth before 37 weeks of gestation.

Patient with at least one associated malformation were classified as having multiple congenital anomalies. Every malformation was referred to its anatomical system: cardiovascular, central nervous, digestive, urinary and genital, musculo-skeletal, or other. They were each qualified as minor or major. Major malformation was defined as a life-threatening malformation with predictable long-term morbidity and/or inability to correct without residual morbidity. All other malformations were classified as minor. Discrimination between locations of measurement of the omphalocele (omphalocele diameter versus size of abdominal wall defect, i.e., neck) was made when mentioned. Based on the literature, we set two thresholds to “define” large omphaloceles at 50 and 80 mm, for both, sac diameter and neck diameter.

To calculate omphalocele prevalence, the official Swiss open access database was used [17]. Official database provide an estimation of annual number of pregnancies from 2000 to 2004 and established numbers since 2004 [17]. A linear regression was performed to calculate interrupted pregnancies from 1998 to 1999. Total number of pregnancies amounted to 454,930 over the study period. There was a total number of 381,700 LB over the study period. Calculation of total prevalence was based on LB, TOPFA and still birth.

The study was approved by the local ethics committee (registration number 2018-02130).

We presented categorical variables as absolute values and percentages, and continuous variables as median and interquartile range (IQR). Statistical comparison was performed using Chi^2^ and Fisher’s exact test, with a *p* value < 0.05 considered significant.

## Results

**Fig. 1:**
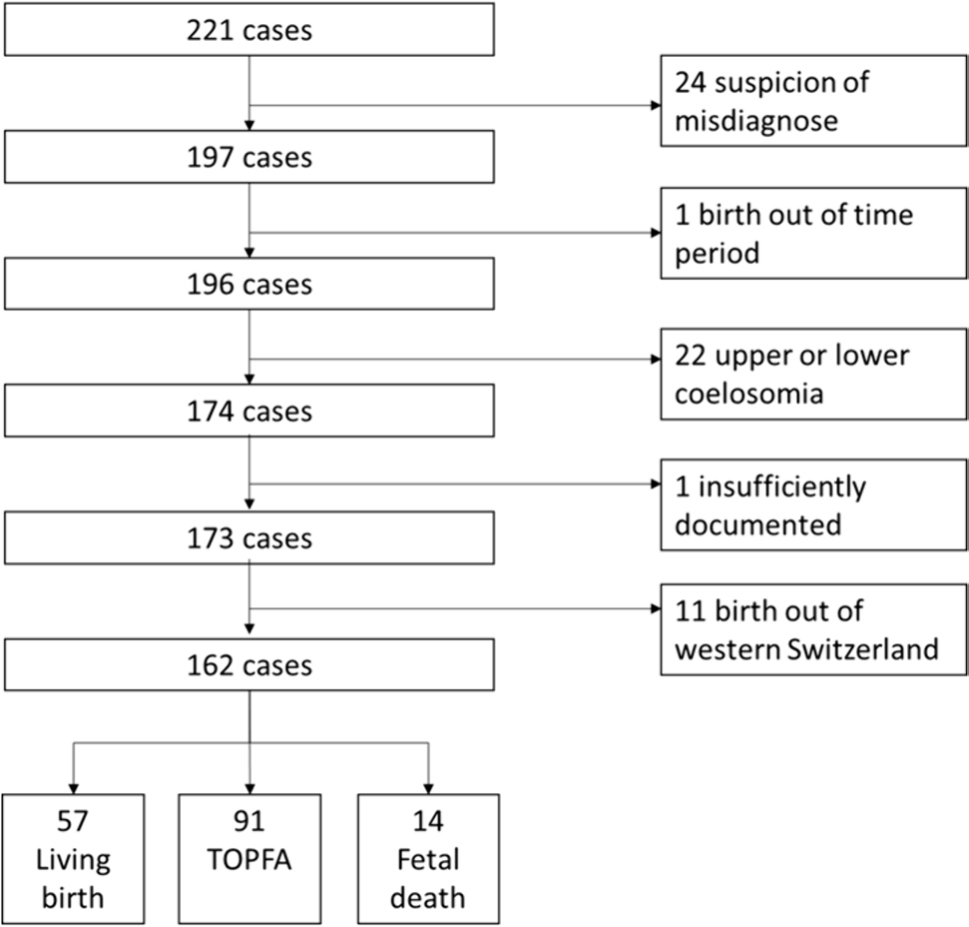
Flow chart of included and excluded patients

We included 162 cases of omphalocele born in Western Switzerland over the studied period (Fig. 1). Of these, 57 (35%) were LB, and the remaining 105 (65%) were non-live births, consisting of 91 (56%) TOPFA and 14 (9%) FD. We found no mention of terminations of pregnancies for maternal reasons. All abortions were classified as TOPFA when medical intervention occurred, or FD if spontaneous.

Over the study period, total prevalence of omphalocele was 3.6 cases per 10,000 births (LB, TOPFA and FD), while LB prevalence was 1.5 cases per 10,000 LB.

## Patient characteristics

The median gestational age at delivery for LB was 38 (IQR 36–38) weeks of gestation, with 13 (30%) being born before 32 weeks. The overall median gestational age at termination of pregnancy was 14 (IQR 13–16) weeks. TOPFA was performed at a median gestational age of 13 (IQR 13–16) weeks, while FD occurred at a median gestational age of 15 (IQR 13–15) weeks. The mean morphologic echography was performed at 16.5 weeks of gestation (IQR 13–19) for LB cases, whereas for TOPFA cases, it was conducted at 12 weeks of gestation (IQR 12–13).

Table 1 present descriptive data for the entire population, and for LB and TOPFA separately:

**Table 1:** Patient characteristics including congenital anomalies described by type, system, and grade

	Total *N* = 162	LB *N* = 57	TOPFA *N* = 91
Male	75 (61%)^a,b^	37 (66%)^b^	36 (58%)^a^
Female	47 (39%)^a,b^	19 (34%)^b^	26 (42%)^a^
Isolated	40 (25%)	18 (32%)	13 (14%)
Multiple congenital anomalies	90 (56%)	37 (65%)	49 (54%)
Major malformation	62 (38%)	13 (23%)	46 (51%)
Genetic anomaly	71 (44%)	13 (23%)	56 (62%)
Trisomy 13,18,21	56 (35%)	6 (11%)	49 (54%)
Syndromic	62 (38%)	9 (16%)	52 (57%)
**Any malformation**			
None	72 (44%)	20 (35%)	42 (46%)
Minor	28 (17%)	24 (42%)	3 (3%)
Major	62 (38%)	13 (23%)	46 (51%)
**Cardiovascular system**			
None	109 (67%)	30 (53%)	67 (74%)
Minor	27 (17%)	22 (39%)	4 (4%)
Major	26 (16%)	5 (8%)	20 (22%)
**Central nervous system**			
None	130 (80%)	48 (84%)	70 (77%)
Minor	6 (4%)	3 (5%)	3 (3%)
Major	26 (16%)	6 (11%)	18 (20%)
**Digestive system**			
None	149 (92%)	48 (84%)	87 (96%)
Minor	5 (3%)	4 (7%)	1 (1%)
Major	8 (5%)	5 (9%)	3 (3%)
**Genito-urinary system**			
None	134 (83%)	40 (70%)	80 (88%)
Minor	13 (8%)	12 (21%)	1 (1%)
Major	15 (9%)	5 (9%)	10 (11%)
**Musculo-skeletal system**			
None	142 (88%)	51 (89%)	78 (86%)
Minor	4 (2%)	2 (4%)	2 (2%)
Major	16 (10%)	4 (7%)	11 (12%)
**Other**			
None	149 (92%)	53 (92%)	83/91 (91%)
Minor	10 (6%)	2 (4%)	7/91 (8%)
Major	3 (2%)	2 (4%)	1/91 (1%)

Total = Termination of pregnancy due to fetal anomaly (TOPFA) + fetal death (FD) + living birth (LB). ^a^ 39 missing data; ^b^ 1 sexual variation

### *LB population (N* = *57)*

Male phenotype represented two thirds of cases. A third had isolated omphalocele. Genetic anomalies were present in about a quarter of patients, of which about half of them had a trisomy 13, 18 or 21. About one of six patients had a syndrome. About two thirds of LB patients had one or more associated malformations. They were mostly of the minor type, and mainly of cardiovascular origin, such as atrial septal defect. If major malformations were present, they were mainly of the nervous, digestive and musculo-skeletal system.

### *TOPFA population (N* = *91)*

Isolated omphaloceles was observed in one of seven fetuses. Genetic anomalies were seen in almost two thirds of cases, of which 88% had a trisomy 13, 18 or 21. Syndromic cases represented 57% of the TOPFA population. 29 (32%) cases were associated with a syndrome or genetic anomalies without structural or anatomical malformations: 18 with Edwards syndrome, four with Down syndrome, two with Patau, two with Turner syndrome, two had sporadic genetic mutations and one had a Beckwith-Wiedemann syndrome. More than half had major-associated malformations. They were mainly from major cardiovascular origin, such as tetralogy of Fallot. There were more major malformations than minor in every system.

## Omphalocele characteristics

### *LB subpopulation* (*N* = 57)

The liver was involved in 37% (17/46) of omphaloceles. 60% (25/42) of patients had a sac diameter greater than 50 mm and 24% (10/42) greater than 80 mm. Measurement on the neck of the sac showed 15% (5/32) larger than 50 mm and 5% (2/32) larger than 80 mm.

## *TOPFA subpopulation* (*N* = 91)

The liver was involved in 68% (13/19) of omphaloceles. Size of the defect and liver involvement is not reported due to too many missing data.

## Comparative analysis of omphalocele size and associated conditions

Table 2 shows the cross table for different characteristics of omphalocele and the patient’s associated conditions in the LB subpopulation. There was no statistical significance for any variable, i.e., nor the size of the omphalocele (with cut-off of 50 mm and 80 mm), nor the liver involvement were associated with more, or less, associated malformations, genetic or chromosomal anomalies.

**Table 2:** Comparative analysis between the size of the omphalocele, the liver involvement and associated malformations for the living birth subgroup

	50 mm *N* = 42 (74%)			80 mm *N* = 42 (74%)			Liver *N* = 46 (81%)		
	< 50 mm *N* = 17	> 50 mm *N* = 25	*p*	< 80 mm *N* = 32	> 80 mm *N* = 10	*p*	Not involved *N* = 29	Involved *N* = 17	*p*
Isolated omphalocele^a^	5 (29%)	5 (20%)	0.7	8 (25%)	2 (20%)	1	9 (31%)	2 (12%)	0.2
Multiple associated malformations	11 (65%)	19 (76%)	0.4	22 (69%)	8 (80%)	0.7	18 (62%)	15 (88%)	0.09
Minor associated malformations	7 (41%)	15 (60%)	0.2	16 (50%)	2 (20%)	0.7	12 (41%)	11 (65%)	0.1
Major associated malformations	4 (24%)	4 (16%)	0.7	6 (19%)	6 (60%)	1	6 (21%)	4 (24%)	1
Genetic anomaly	3 (18%)	6 (24%)	0.7	7 (22%)	2 (20%)	1	7 (24%)	3 (18%)	0.7
Trisomy 13, 18, 21	1 (6%)	2 (8%)	1	3 (9%)	0 (0%)	1	4 (14%)	0 (0%)	0.3
Syndromic	2 (12%)	4 (16%)	1	6 (19%)	0 (0%)	0.3	6 (21%)	0 (0%)	0.07
Cardiovascular system anomaly	10 (59%)	13 (52%)	0.7	17 (53%)	6 (60%)	1	12 (41%)	11 (65%)	0.1
Central nervous system anomaly	1 (6%)	3 (12%)	0.6	3 (9%)	1 (10%)	1	4 (14%)	2 (12%)	1
Digestive system anomaly	1 (6%)	6 (24%)	0.2	5 (16%)	2 (20%)	1	6 (21%)	2 (12%)	0.7
Genito-urinary system anomaly	5 (29%)	8 (32%)	0.8	10 (31%)	3 (30%)	1	9 (31%)	5 (29%)	0.9
Musculo-skeletal system anomaly	2 (12%)	2 (8%)	1	3 (9%)	1 (10%)	1	2 (7%)	2 (12%)	0.6
Other anomaly	0 (0%)	3 (12%)	0.3	1 (3%)	2 (20%)	0.1	2 (7%)	1 (6%)	0.9

^a^Isolated omphaloceles defined by the absence of anatomical, genetic, or syndromic abnormalities

## Discussion

This analysis of a large cohort of omphaloceles patients, including prenatal data, confirms as expected that the proportion of cases resulting in TOPFA was higher among fetuses with major malformations, genetic or chromosomal anomalies. Due to the high rate of early terminations, echographic assessments for malformations were conducted differently between LB and TOPFA populations. Although all cases underwent confirmation through serial echography, this difference may have led to an underestimation of the rate and severity of malformations in the TOPFA population. Yet, and in contrary to previous publications, we show that the size of the omphalocele and/or liver involvement does not allow for conclusions regarding the presence of number of associated malformations, genetic or chromosomal anomalies.

## Epidemiology

Our study found a total prevalence of 3.6/10,000 birth (live and still), which is consistent with previous literature reporting total prevalence rates of 2.1–3.8/10,000 births [2, 12, 14, 26–30]. The LB prevalence of 1.5/10,000 is also consistent with the literature, which reported LB prevalence rates of 1.3–1.92/10,000 live births [3, 12, 26, 28, 29, 31–33].

In our cohort, the proportion of isolated omphaloceles, associated anatomical malformations, and genetic abnormalities aligns with previous reports, except for central neural system anomalies, where we found that they had a higher incidence when compared to other publications (see Table 3). This discrepancy may be attributed to our study’s broad inclusion criteria, which encompassed a wide range of neural anomalies, including hydrocephaly and holoprosencephaly, rather than only neural tube defects.

**Table 3:** Comparison of the present study with the literature of omphalocele characteristic and congenital anomalies by system

	Stoll et al. [14]*	Springetti et al. [29]*	Raymond et al. [34]**	Conner et al. [33]*,+	Nembhart et al. [12]*,+	Benjamin et Wilson [19]*	Present study		
							Total	LB	TOPFA
*N*	58	671	274	42	2499	814	162	57	91
Isolated omphalocele^a^	26%	37%	25%	33%	37%	20%	25%	32%	14%
Multiple congenital anomalies	40%	31%	73%	54%	42%	80%	56%	65%	54%
Genetic anomaly	34%	32%	15%	29%	21%	14%	44%	23%	62%
Cardiovascular system anomaly	13%	17%	56%			23%	33%	47%	26%
Central nervous system anomaly	7%	14%	5%			8%	20%	16%	23%
Digestive system anomaly	13%	8%	15%			6.8%	8%	16%	4%
Genito-urinary system anomaly	24%	12%	18%			21%	17%	30%	12%
Musculo-skeletal system anomaly	17%	12%	18%			24%	12%	11%	14%

Total = Termination of pregnancy due to fetal anomaly (TOPFA) + Fetal death (FD) + living birth (LB). ^a^Isolated omphaloceles defined by the absence of anatomical, genetic, or syndromic abnormalities

*Proportion of total population; **proportion of LB population; +no repartition of malformation types available

As expected, in the TOPFA population, we observed a higher rate of major congenital malformations compared to the LB population. The TOPFA population also showed compared to the LB population a higher occurrence of genetic malformations and more cases were categorized as syndromic even when no structural anomalies are apparent. These findings support the general thought that the higher morbidity an omphalocele case shows, the more the pregnancy is terminated.

## Prognostic criteria for omphalocele

Formerly, no distinction was made between the different abdominal wall defects. In the 1960ies Duhamel initiated the distinction between omphalocele and laparoschisis [9]. From then on, some associations between the size of omphalocele and associated malformations, and thus prognosis, have been proposed, but debates still persist [24, 25]. The development of prenatal ultrasound in the 70 s allowed the characterization of congenital anomalies in the prenatal period. Following the congenital diaphragmatic hernia model, some authors have looked for quantifiable prognostic factors in the prenatal period [23].

In 1989, M.D. Hughes became interested in the ultrasound factors of omphalocele predictive of mortality [35]. He notes that in his cohort of 43 fetuses, fetal mortality is significantly associated with associated malformations but not with the absolute size of the omphalocele. To rationalize the measurement of omphalocele, a ratio was suggested by measuring the maximum diameter of the omphalocele sac and the transverse abdominal diameter at the umbilical vein (OC/AD), however, no statistical correlation could be found. In 2011, F.J. Montero was interested in prenatal ratios such as the size of the omphalocele along the length of the femur (OC/Fl), abdominal circumference (OC/AC) or head circumference (OC/HC) as a predictor of primary closure [23]. He compared them to different definitions of giant omphalocele in their ability to predict primary closure. He concluded that the ratios are superior to absolute definitions of giant omphaloceles to predict closure in a single time. The OC/HC > 0.21 ratio seemed to be the best predictor of tiered closure. The same year, C.E. Kleinrouweler found that the OC/AC ratio was correlated with the type of surgery and added a correlation with the risk of respiratory failure and hepatic hernia [22]. However, OC/AC could not distinguish between isolated, genetic or syndromic omphalocele. The correlation between viscero-abdominal disproportion and the type of surgery seemed to be confirmed through the studies of J.A. Fawley and N.C.J Peters with, however, different cut-offs [36, 37]. This said, we realized by performing our study that such data are difficult to obtain, and the great majority of our prenatal ultrasounds did not report data to calculated ratios. It seems that in practice, measurements are not carried out systemically.

The idea behind a classification is to predict morbidity and outcome. It rather seems that in daily practice of pediatric surgery as well as in scientific publications omphaloceles are classified based on size only. However, to now, the only established predictors for outcome are the presence of associated anatomical or genetical anomalies. This said, in recent years, many authors proposed some correlations between measurement and predictive element of outcomes: Raymond et al. proposed in 2019 a retrospective study of the largest contemporary cohort of neonates including 274 cases of omphalocele born alive, addressing the question if the clinical outcome differed according to the size of the omphalocele [34]. The definition used of large omphalocele was based either on the clinical evaluation of the surgeon mentioning a large or giant omphalocele or a measurement greater than 5 cm of the defect. The study revealed that these combined definitions of large omphalocele were an independent predictor of outcome in terms of length of stay, intensive care unit hospitalization, mechanical ventilation, tracheostomy, time of parenteral nutrition use and decrease in overall survival. In 2008, Van Eijck reported differences between large (more than 5 cm with hepatic content) and minor omphaloceles in initial care, but that long-term quality of life does not seem to be impacted by the size of the omphalocele [38]. In a retrospective study of 2015, where large omphalocele was defined as greater than 4 cm, Kumar et al. brings several new elements: the authors reveled differences in associated malformations according to the sex and size of the omphalocele, with a small omphalocele being preferentially associated with more gastrointestinal malformations and less heart defects [25]. In contrast, our retrospective study of also a rather large cohort showed no clear trend of association between classical classification criteria, gender, and associated malformations, genetical anomalies or syndromes.

Despite many publications over decades on this topic, literature provides conflicting information on omphalocele classification and their associated morbidity. It seems that a general standard classification is lacking and that the scientific community defines “giant” and “large” and “minor” to their liking. Moreover, as cut-off size and location of measurement change from on publication to another, it is difficult to do any comparisons between cohorts. Thus, we do believe that pediatric surgeons and obstetricians should join their efforts to agree on a standardized classification, allowing for future research and harmonized conclusions.

## Limitations

Limitations of this current study lie in its retrospective nature. As included cases derived from different databases and as prenatal and later aborted fetuses were sometimes not completely documented. But fortunately, the cohort was of rather large size, which partly counteracted this limitation. In addition, some cases born in the investigated territories of Switzerland could have been treated anywhere else in Switzerland and therefore be missing in our analyses.

## Conclusions

Our study confirms that the proportion of omphalocele cases resulting in TOPFA was higher among fetuses with major malformations, genetic or chromosomal anomalies. Yet, our study contrasts with the literature on the relationship between omphalocele size and associated conditions: in our cohort, absolute size of the omphalocele at birth was not correlated to any associated anatomical, chromosomal or syndromic condition.

## Data Availability

No datasets were generated or analyzed during the current study.

## Abbreviations

AD: Abdominal diameter
FD: Fetal death
FL: Femur length
HC: Head circumference
IQR: Interquartile range
LB: Live births
OC: Omphalocele
OEIS complex: Omphalocele-cloacal exstrophy-imperforate anus-spinal defect complex
TOPFA: Termination of pregnancy due to fetal anomaly
